# Attenuation of intestinal ischemia-reperfusion-injury by anesthetics: a potentially protective effect of anesthetic management in experimental studies

**DOI:** 10.3389/fphar.2024.1367170

**Published:** 2024-02-20

**Authors:** Zhan Huang, Yiping Bai, Ying Chen, Ye Chen, Yuan Jiang, Jun Zhou

**Affiliations:** ^1^ Department of Anesthesiology, The Affiliated Hospital, Southwest Medical University, Luzhou, China; ^2^ Anesthesiology and Critical Care Medicine Key Laboratory of Luzhou, Southwest Medical University, Luzhou, China; ^3^ Department of Anesthesiology, Dazhou Integrated TCM & Western Medicine Hospital, Dazhou Second People’s Hospital, Dazhou, China; ^4^ Department of Traditional Chinese Medicine, The Affiliated Hospital, Southwest Medical University, Luzhou, China; ^5^ Clinical Medical College and The First Affiliated Hospital of Chengdu Medical College, Chengdu, China

**Keywords:** ischemia-reperfusion injury, anesthetic management, gas anesthetics, intravenous anesthetics, analgesics

## Abstract

Intestinal ischemia-reperfusion injury (IRI) is a potentially severe clinical syndrome after major surgical procedures. In addition to causing intestinal mucosa injury, intestinal IRI further damages distant organs, causing the severity of the condition in patients. So far, effective therapy for intestinal IRI is still absent, and the survival rate of the patients is low. Previous experimental studies have shown that some anesthetics can alleviate intestinal IRI and protect organs while exerting their pharmacological effects, indicating that reasonable perioperative anesthesia management may provide potential benefits for patients to avoid intestinal IRI. These meaningful findings drive scholars to investigate the mechanism of anesthetics in treating intestinal IRI in-depth to discuss the possible new clinical uses. In the present mini-review, we will introduce the protective effects of different anesthetics in intestinal IRI to help us enrich our knowledge in this area.

## Introduction

Intestinal ischemia-reperfusion injury (IRI) is a potentially severe clinical syndrome of several major surgical procedures, including cardio-pulmonary bypass surgery, liver transplantation, bowel resection and transplantation, abdominal aortic aneurysm surgery, and strangulated hernias (Yasuhara, 2005; Abboud et al., 2008; Nickkholgh et al., 2013). Intestinal IRI is a serial cascade of pathophysiologic changes of mucosal barrier failure, bacteria translocation, and inflammation caused by the disruption of blood and oxygen supply and the subsequent reperfusion of the intestine after arterial obstruction, venous thrombosis, and diffuse vasospasm (Shen et al., 2014). The intestine is more sensitive to IRI, which damages the integrity of the intestinal mucosal barrier and may lead to bacterial translocation through the intestinal wall, peritonitis, and subsequent systemic inflammatory response syndrome (SIRS) (Collard and Gelman, 2001; Cheng et al., 2013). Intestinal IRI can also cause damage to distant organs, especially acute lung injury (ALI), and may even lead to multiple organ dysfunction syndrome (MODS), endangering the patient’s life (Li et al., 2020; Liao et al., 2022). Therefore, how to attenuate intestinal IRI after major surgical procedures encourages scholars to focus on exploring effective and safe prevention and treatment methods. Although several therapeutic strategies for intestinal IRI have been reported so far, including energy therapy, anti-free radical therapy, anti-leukocyte adhesion therapy, glucocorticoids, and mesenchymal stem cells (MSCs) therapy, and even some miRNAs are considered new targets for the research and development of innovative drugs, there is no definite ideal treatment available for it (Akbari, 2020; Liao et al., 2022; Shi et al., 2022).

In recent years, many studies have demonstrated that some anesthetics attenuate operative stress during the perioperative period and have organ protective effects while exerting their pharmacological effects, particularly against IRI in various organs, attracting extensive attention and reflection (Erturk, 2014; Álvarez et al., 2014; Motayagheni et al., 2017). In addition to ischemic preconditioning (IPC) and remote ischemic preconditioning (RIPC), reasonable anesthetic management in the perioperative period may be another strategy to supply some benefits for patients to avoid intestinal IRI. Some scholars have attempted to select specific anesthetics from gas anesthetics, intravenous anesthetics, analgesics, and sedatives to attenuate intestinal IRI and further explore the underlying mechanism. In particular, the research results of sevoflurane, propofol, dexmedetomidine, and remifentanil have provided experimental evidence of attenuation of intestinal IRI, suggesting that they may represent a prioritized selection of anesthetic management. In the present mini-review, we will introduce the protective effects of different anesthetics in intestinal IRI to help us enrich our knowledge in this area.

## Sevoflurane

Sevoflurane is a widely used inhalation anesthetic in clinical practice, which has the advantage of inducing rapid and rapid resuscitation compared with other inhalation anesthetics (Duffy and Matta, 2000; Liang et al., 2021). Sevoflurane is potentially neurotoxic and may cause weak effects on cognitive function after a short or single exposure and cognitive dysfunction after prolonged or repeated exposure. It raises our concern about the potential neurotoxic effect of sevoflurane, particularly in brain development in childhood (Sun et al., 2022). However, existing findings demonstrated that sevoflurane has the activity of anti-oxidative stress, anti-inflammation, and neuroprotective effects and also can attenuate IRI, for instance, cerebral IRI injury (Liang et al., 2021; Lyu and Li, 2023; Xing et al., 2024). Hei and his research team first explore the protection effect of sevoflurane preconditioning on small intestinal IRI in Sprague-Dawley (SD) rats. In their study, the SD rats were first exposed to 2.3% sevoflurane for 1 h per day for 3 days. Subsequently, they clamped the rats’ superior mesenteric artery (SMA) for 75 min and then released the clamp to maintain reperfusion for 2 h. They found no difference in the survival rates between the rats treated with sevoflurane preconditioning or oxygen alone. However, a degree of intestine injury and Chiu’s scores of the rats treated with sevoflurane decreased obviously. Sevoflurane preconditioning also downregulated the myeloperoxidase (MPO) activities, ICAM-1 protein expression, and IL-6 concentrations, indicating sevoflurane possibly through inhibiting neutrophil sequestration and systemic inflammation to attenuate small intestinal IRI. They suggested that sevoflurane preconditioning may provide some benefits in alleviating postoperative intestinal ischemia and mortality (Gan et al., 2013). They also confirmed that sevoflurane preconditioning protects SD rats from intestinal ischemia-reperfusion-induced ALI via inhibiting NADPH oxidase and the synergistic action between oxidative stress and mast cell activation in a subsequent study, which may have a positive meaning for the clinical treatment of IIR-mediated ALI (Luo et al., 2015). Liu et al. have successively studied and reported on the effects of clinically relevant concentrations of sevoflurane in attenuating intestinal IRI. They first exposed the intestinal IRI rat models to 0.25, 0.5, and 1.0 minimum alveolar concentration (MAC) sevoflurane before, during, or after intestinal ischemia-reperfusion, respectively. They observed that 0.5 MAC sevoflurane can reduce epithelial apoptosis for protecting the intestinal mucosa without severe respiratory inhibition during ischemia-reperfusion. However, the protective effect can be partially reversed by phosphatidylinositol 3 kinases (PI3K) inhibitor LY294002, suggesting that sevoflurane inhibits intestinal mucosal epithelial apoptosis via the activation of the PI3K/Akt pathway (Liu et al., 2015a). In the subsequent research, they observed the changes in protein kinase C (PKC) and mitochondrial ATP-sensitive potassium channel (mKATP) in intestinal IRI rats treated with sevoflurane preconditioning, suggesting that the protecting effects of sevoflurane preconditioning on intestinal IRI was also dependent on the activation of PKC and mKATP (Liu et al., 2015b). Later, they found that sevoflurane preconditioning reversed the high expression of NF-κB P65 protein, proinflammatory cytokine tumor necrosis factor-α (TNF-a), and interleukin-6 (IL-6) and upregulated PPARγ protein in the intestinal mucosa of intestinal IRI rats, indicating that sevoflurane inhibits the intestinal inflammatory reaction via activation of the PPARγ/NF-κB pathway (Liu et al., 2020). Notably, they compared the efficacy of sevoflurane preconditioning and IPC attenuating intestinal IRI while studying the potential mechanism (Luo et al., 2015a). The results of the histopathological test and Chiu’s scores showed both sevoflurane preconditioning and IPC attenuated intestinal injury in rats, indicating that sevoflurane preconditioning can provide similar anti-intestinal IRI effects as IPC. These findings provide valuable experimental evidence of clinically relevant concentrations of sevoflurane in attenuating intestinal IRI, which may have great translational potential in patients at risk of intestinal IRI.

## Propofol

Propofol, as a commonly used short-acting intravenous anesthetic, has some advantages in the anesthetic management of the perioperative period, including fast onset of anesthesia, short recovery time, repeated administration, and antiemetic effect. Hence, propofol has been widely administered for the induction and maintenance of anesthesia so far (Lundström et al., 2010; Walsh, 2022). Previous studies have shown that propofol also has anti-inflammatory, antioxidant, and immunomodulatory properties, contributing to its neuroprotective effects but even affecting the cancer prognosis (Marik, 2005; Kotani et al., 2008; Gao et al., 2020). Given the unique pharmacological effects of propofol, many scholars have investigated the effect of propofol on intestinal IRI. Liu et al. selected three propofol regimens to investigate the effect on intestinal mucosal injury after intestinal IRI. Wistar rats were treated with propofol at a sedative dose before, during, or after the intestinal IRI, respectively. The histological measurement of intestinal mucosal injury and Chiu’s scores showed that three different propofol treatment regimens significantly alleviated intestinal mucosal injury after intestinal IRI, especially propofol preconditioning displayed the best protective effect. Propofol preconditioning can inhibit the levels of lipid peroxidation product malondialdehyde (MDA) via attenuating nitric oxide (NO) and endothelin-1 (ET-1) production and stimulate an over-production of endogenous superoxide dismutase (SOD) activity in the intestinal mucosa, indicating that propofol attenuates intestinal IRI may be attributable to its antioxidant properties. They suggested that propofol preconditioning at a sedative dose provides a profound protective effect in intestinal IRI rats, and it is worth exploring the clinical translational potential of propofol in patients at risk for intestinal IRI following major cardiac surgery or for critical care (Liu et al., 2007). In addition to inhibiting MDA in intestinal IRI rats, Kaplan et al. (2007) found that propofol can also inhibit the production of inflammatory cytokines, such as tumor necrosis factor-alpha (TNF-a) and interleukin-6 (IL-6). They suggested that the anti-inflammatory and antioxidant properties may contribute to the protection of propofol in intestinal ischemia/reperfusion-induced liver injury. Vasileiou et al. (2012) believed those properties also seem to be the crucial mediating mechanisms of propofol for efficiently preventing intestinal ischemia/reperfusion-induced lung injury. Li et al. (2021a) confirmed that the anti-inflammatory of propofol comes from downregulating the p38 MAPK/NF-κB signaling pathway to inhibit the production of inflammatory cytokines in intestinal IRI rats. Wu et al. (2020) observed propofol decreased the number of cell apoptosis in the intestinal tissue of intestinal IRI rats besides anti-inflammatory and antioxidant. Liu et al. (2008) attributed this anti-apoptotic effect of propofol preconditioning to its antioxidant property modulating the ceramide pathway. Some studies proved mucosal mast cell (IMMC) activation is critical in intestinal IRI by secreting many mediators to induce intestinal epithelial injury and integrity disruption. Propofol preconditioning can suppress IMMC activation, and it can explain why propofol can attenuate Intestinal IRI, restore intestinal epithelial cell integrity, and prevent intestinal IRI-induced lung injury in rodents and even pigs from other perspectives (Zhao et al., 2014; Gan et al., 2015; Bian et al., 2021; Li et al., 2022a). The above findings showed propofol may provide a meaningful anesthetic management regimen for preventing intestinal IRI and organ injury following major surgery and is worthy of a further clinical study to examine the clinical significance.

## Dexmedetomidine

Dexmedetomidine (DEX) is a highly selective α2-adrenoceptor agonist with unique sedative and analgesic properties and widespread use in the perioperative period (Cai et al., 2022). Previous studies have demonstrated that DEX also has anti-inflammatory and anti-apoptotic properties, allowing it to provide multiple organ-protective effects in animal models of IRI (Cai et al., 2014; Zhao et al., 2022). Zhang et al. compared the effects of different doses of DEX given 1 h before intestinal ischemia or 1 h after the beginning of reperfusion on the intestinal injury of rats. They found that DEX at 2.5 μg/kg/h has no beneficial effects before or after ischemia, while DEX at 10 μg/kg/h led to severe hemodynamic suppression. Only when DEX at 5 μg/kg/h was infusion before ischemia can decrease intestinal injury and rat mortality by inhibiting the inflammatory response and intestinal mucosal epithelial apoptosis via α2 adrenoreceptor activation. Notably, the dose of 5 μg/kg/h DEX used in rats is equal to approximately 0.8 μg/kg/h in humans, which is a safe dose to apply in cardiovascular surgery and the ICU. However, DEX lacks a protective effect after ischemia. They speculated that this ineffectiveness may due to the slow onset of dexmedetomidine, which reaches its effect approximately 15 min after intravenous administration (Zhang et al., 2012). Some scholars also suggested this ineffectiveness may be due to DEX not inhibiting JAK/STAT signaling after ischemia, and the JAK/STAT signaling regulates the signal transduction for various cytokines and growth factors in inflammation processes and plays a pivotal role in intestinal IRI (Zhang et al., 2020). Another study also demonstrated the intestinal protection of DEX preconditioning in intestinal IRI rats. The authors suggested that DEX has good free radical scavenging and antioxidant properties, anti-apoptotic effects, and anti-inflammatory effects during the progress of intestinal IRI (Zhang et al., 2015). Shen et al. (2013) further evaluated the protective effect of DEX’s anti-inflammatory on intestinal IRI-induced lung injury, and they suggested the inhibitory effect of DEX on cytokine production and the immune response in lung tissue via modulating the TLR4/MyD88 pathway may provide valuable and effective protection to intestinal IRI rats. Chen et al. (2020a) suggested that the cannabinoid receptor CB2-mediated PI3K/Akt pathway is also involved in the function of DEX against lung injury in intestinal IRI rats. Interestingly, Li et al. (2022b) suggested that the anti-inflammatory effect of DEX contributes to attenuating early cognitive dysfunction induced by intestinal IRI mice, indicating that DEX may also provide some benefits in reducing the incidence of cognitive dysfunction. Some new protective mechanisms of DEX in intestinal IRI in animal models have been reported in recent years. DEX can attenuate intestinal I/R injury by decreasing ferroptosis and pyroptosis, enhancing mitophagy, promoting the mitochondrial localization of TERT, and microbiota-related mechanisms (Liu et al., 2021; Zhang et al., 2021; Dong et al., 2022; Hu et al., 2022; Hou et al., 2023). These findings provide new experimental evidence supporting the protective effect of DEX against intestinal IRI and a unique insight into the clinical use of DEX, which has positive significance for DEX administration in the perioperative period.

## Remifentanil

Remifentanil is a short-acting opioid with high analgetic potency, widely used in intra-operative or postoperative analgesia. It also takes part in general anesthesia induction and maintenance as a component of total intravenous anesthesia (Grape et al., 2019; Sridharan and Sivaramakrishnan, 2019; Ren et al., 2022). Remifentanil can attenuate the IRI of organs through multiple mechanisms, such as anti-inflammatory, antioxidant, and anti-apoptotic signaling pathways, that have attracted the attention of scholars (Yi et al., 2023). Some scholars have confirmed the protective effects of remifentanil on intestinal IRI in their studies. Cho et al. (2013) injected 1 μg/kg of remifentanil into C57BL/6J mice before clamping the SMA for 30 min and then 60 min of reperfusion. The tissue injury and lipid peroxidation of jejunum and ileum and systemic IL-6 were analyzed by histology, malondialdehyde (MDA), and ELISA, respectively. They found that remifentanil preconditioning can attenuate the intestinal IRI and inhibit lipid peroxidation and systemic inflammatory response, indicating that pre-treatment of remifentanil may bring potential benefits in the clinical prevention of intestinal IRI. Shen et al. (2016) used the rat models and the rat intestinal epithelial IEC-6 cells to evaluate the protective effects of remifentanil preconditioning. They found that remifentanil preconditioning attenuated intestinal injury in intestinal IRI rats and IEC-6 cell apoptosis after being subjected to oxygen and glucose deprivation (OGD), but naltrindole (a δ-OR selective antagonist) and CTOP (a μ-OR selective antagonist) can markedly attenuate these changes. They suggested that δ- and μ-opioid receptors may play a critical role in the protection against intestinal IRI of remifentanil preconditioning. Then, they proposed a new perspective to explain the mechanism of remifentanil preconditioning in protection against intestinal IRI in a recent study. They found that remifentanil preconditioning attenuated intestinal IRI by reducing oxidative and ER stress, and the PDIA3 gene played an essential role in this protection process, but p38MAPK inhibitor (SB203580) can suppress the PDIA3 expression and abolish the intestinal protection of remifentanil, indicating that remifentanil activates p38MAPK to PDIA3 expression for inhibiting intestinal IRI-induced oxidative and ER stress (Shen et al., 2022). In addition to the anti-inflammatory and antioxidant activity, Sayan-Ozacmak et al. (2015) suggested that remifentanil preconditioning can also improve intestinal contractility in intestinal IRI rats, resulting in restoring dysfunction of intestinal motility induced by the IRI. Therefore, the protection mechanism of remifentanil in intestinal IRI may be multifactorial, and the exact mechanism still needs further elucidation.

## Other anesthetics

Ketamine is often used for anesthesia, analgesia, and sedation, but its applications beyond anesthesia are involved in the treatment of addiction, depressive episodes, asthma, and even inhibit cancer growth (Ivan Ezquerra-Romano et al., 2018; Nowacka and Borczyk, 2019; Mihaljević et al., 2020; Ritter et al., 2020; Adegbola et al., 2023). In addition, ketamine can also protect various tissues from IRI (Xiao et al., 2012; Li et al., 2018). Previous studies found that pre-treatment of ketamine can reduce inflammatory cell infiltration and intestinal injury in intestinal IRI rats (Cámara et al., 2008; Guzmán-De la Garza et al., 2010a; Guzmán-De la Garza et al., 2010b). Ketamine also has some anticoagulant and platelet anti-aggregation properties and can improve the intestinal transit delay, contributing to the protective effects of ketamine. Guzmán-De la Garza et al. (2010b) suggested that an intact enteric nervous system seems to need in the protective action of ketamine in intestinal IRI rats. Parecoxib sodium is an injectable COX-2-specific inhibitor and usually used for postoperative analgesia (Cheer and Goa, 2023). Li et al. reported that parecoxib sodium also exerts protective effects in intestinal IRI rats. They found that parecoxib sodium preconditioning attenuated intestinal injury and increased the rat survival rate by inhibiting inflammation, oxidative stress, and apoptosis (Li and Zheng, 2021). Moreover, Clarysse et al. (2023) observed that isoflurane has anti-inflammatory effects and can reduce intestinal epithelial damage and permeability in intestinal IRI rats, and combined with oxygen-supplementation will provide additional benefits in the attenuation of intestinal IRI, indicating that anesthetic management will bring substantial positive influence in a rodent model of intestinal IRI.

## Summary and outlook

Intestinal IRI causes severe intestinal mucosa histopathological injury and further damage to distant organs, causing the severity of the condition in patients. Unfortunately, effective therapy for intestinal IRI is still absent, leading to a high rate of mortality. According to statistics, about 26% of patients unable to live for more than a year (Wang et al., 2021). Exploring the protection against intestinal IRI of anesthetic management is very important for patients who will suffer major surgical procedures. The research on specific anesthetics introduced above indicates that pre-treatment of these agents confers protection against intestinal IRI and distant organ injury by their anti-inflammatory, anti-oxidant, and anti-apoptotic properties. Preconditioning with specific anesthetics may be advantageous in patients with intestinal IRI, providing a referable opinion for anesthetic management in the perioperative period. However, some limits of current studies need attention and to be discussed.

First, the existing exciting research results are mainly from preclinical studies, but heterogeneity and methodological quality of these research are still unavoidable limitations. Animal experiments serve as a basis for exploring the efficacy and potential mechanisms of the anesthetics in treating intestinal IRI (Hou et al., 2023). The animals commonly used to build animal models of intestinal IRI include rodents, pigs, cats, and dogs (Gonzalez et al., 2015). Previous studies on intestinal IRI mainly employed rodent models (see Table 1). Compared to large animals, rodents have the advantage of low cost, rapid reproduction rate, number of available and well-established models. Wang et al. (2021) summarized the establishment methods of the intestinal IRI rat model from strains, gender, age, weight, anesthesia, surgical details, ischemia and reperfusion time, and perioperative care in their published review. They realized that building a uniform standard for the intestinal IRI rat model would provide a reliable basis for the horizontal comparison of mechanism research. However, there are difficulties in surgery and a high mortality rate in the procedure of building IRI rodent models. In addition, the pathological and physiological changes of the rodent models’ intestinal tract differ to some extent from those in humans. Some scholars employed big animals to build the IRI animal models because it may be easier to develop safe preclinical protocols directly transferable to humans using those animals. For example, pigs are considered the ideal model for human intestinal IRI research because of their approximate morphology and function of human intestines. The bigger size of pigs is the ease of surgical manipulation. Scholars can temporarily occlude the smaller vessels within the mesentery without compromising the integrity and then reperfusion to cause intestinal IRI in pigs. However, the experimental costs, feeding management, and the number of models also limit the conduct of related experiments on intestinal IRI pig models. In addition to the animal models, the appropriate model-making method is critical to experimental design. Gonzalez et al. (2015) classified the intestinal IRI modeling methods as complete vascular occlusion (SMA ligation and SMA embolization), low-flow ischemia, and segmental mesenteric vascular occlusion. They also compared the advantages and disadvantages of these methods in their published review. They suggested that the mesenteric vascular occlusion model of intestinal ischemia may be the prioritized modeling method because it can be readily performed in large animals (e.g., pigs) and rodents. Given current intestinal IRI animal experiments still lack optimal choices in animal species and modeling methods, the methodologic of the preclinical studies about anesthetics still needs to be further well-designed before clinical implication.

**TABLE 1:** Literature examples of anesthetic application in Intestinal IRI animal models.

Anesthetics	Dose	Animal species	IRI modeling method	References
Sevoflurane	2.3%	SD rat	75 min SMA occlusion followed by 120 min reperfusion	Gan et al. (2013), Luo et al. (2015)
Sevoflurane	0.5, 1.0, and 2.0%	SD rat	60 min SMA occlusion followed by 120 min reperfusion	Liu et al. (2015a), Liu et al. (2015b), Liu et al. (2020)
Propofol	50 mg/kg	Wistar rats	60 min SMA occlusion followed by 180 min reperfusion	Liu et al. (2007), Liu et al. (2008)
Propofol	10 mg/kg	Wistar rats	30 min SMA occlusion followed by 120 min reperfusion	Kaplan et al. (2007)
Propofol	60 mg/kg	Wistar rats	45 min SMA occlusion followed by 240 min reperfusion	Vasileiou et al. (2012)
Propofol	50 mg/kg	SD rats	45 min SMA occlusion followed by 90 min reperfusion	Li et al. (2021a)
Propofol	60 mg/kg	SD rats	60 min SMA occlusion followed by 120 min reperfusion	Wu et al. (2020)
Propofol	50 mg/kg	SD rats	75 min SMA occlusion followed by 120 min reperfusion	Zhao et al. (2014), Gan et al. (2015)
Propofol	10 mg/kg/h	miniature pigs	120 min SMA occlusion followed by 240 min reperfusion	Bian et al. (2021)
Dexmedetomidine	2.5, 5, and 10 μg/kg/h	SD rats	60 min SMA occlusion followed by 120 min reperfusion	Zhang et al. (2012)
Dexmedetomidine	10, 20, and 50 μg/kg	Wistar rats	60 min SMA occlusion followed by 120 min reperfusion	Zhang et al. (2020)
Dexmedetomidine	50 μg	Wistar rats	60 min SMA occlusion followed by reperfusion	Zhang et al. (2015)
Dexmedetomidine	2.5 and 5.0 μg/kg/h	SD rats	60 min SMA occlusion followed by 120 min reperfusion	Shen et al. (2013), Liu et al. (2021)
Dexmedetomidine	5.0 μg/kg/h	SD rats	60 min SMA occlusion followed by 120 min reperfusion	Chen et al. (2020a)
Dexmedetomidine	50 μg/kg	C57BL/6J mice	45 min SMA occlusion followed by 24 h reperfusion	Li et al. (2022a)
Dexmedetomidine	10 and 100 μg/kg	Wistar rats	60 min SMA occlusion followed by 60 min reperfusion	Hu et al. (2022)
Dexmedetomidine	400 μg/kg	C57BL/6 mice	60 min SMA occlusion followed by 180 min reperfusion	Dong et al. (2022)
Remifentanil	1.0 μg/kg	C57BL/6 mice	30 min SMA occlusion followed by 60 min reperfusion	Cho et al. (2013)
Remifentanil	0.1, 0.2, 0.6, and 1.0 μg/kg	SD rats	60 min SMA occlusion followed by 120 min reperfusion	Shen et al. (2016)
Remifentanil	1.0 μg/kg	C57BL/6 mice	45 min SMA occlusion followed by 240 min reperfusion	Shen et al. (2022)
Remifentanil	2.0 μg/kg/min	Wistar rats	30 min SMA occlusion followed by 180 min reperfusion	Sayan-Ozacmak et al. (2015)
Ketamine	100 mg/kg	Wistar rats	45 min SMA occlusion followed by 60 min or 24 h reperfusion	Cámara et al. (2008)
Ketamine	6.25, 12.5, 50, and 100 mg/kg	Wistar rats	30 min SMA occlusion followed by 60 min reperfusion	Guzmán-De la Garza et al. (2010a)
Ketamine	50 mg/kg	Wistar rats	30 min SMA occlusion followed by 60 min reperfusion	Guzmán-De la Garza et al. (2010b)
Parecoxib sodium	10 and 20 mg/kg	SD rats	60 min SMA occlusion followed by 120 min reperfusion	Li and Zheng (2021)
Isoflurane	induction with 5% and maintenance with 1.5%–1.75% at 1 L/min	SD rats	60 min SMA occlusion followed by 60 min reperfusion	Clarysse et al. (2023)

Second, the dosage of anesthetics in most intestinal IRI animal experiments is clinically relevant concentration, but there are growing concerns about the safety of anesthetic use. It is well-known that the neurotoxicity of anesthetics may inhibit the development of children’s nervous systems, weaken memory and learning functions, cause postoperative delirium, and even induce long-term cognitive dysfunction (Jungwirth et al., 2009; Kang et al., 2017; Johnson et al., 2019; Johnson et al., 2019; McCann and Soriano, 2019). Research has confirmed that long-term exposure to sevoflurane in young animals could lead to a 50-fold increase in the rate of neuroapoptosis (Sun et al., 2022). Some scholars suggested that anesthetic-induced neuroapoptosis is the leading cause of neurotoxicity, and exposure to anesthetics can cause neuroapoptosis through several different molecular mechanisms. For example, sevoflurane induces neuroapoptosis through the brain-derived neurotrophic factor (BDNF)-modulated apoptotic cascade, mitochondria-mediated apoptosis, death receptor signaling, intracellular ROS, and intracellular calcium homeostasis (Sun et al., 2022). Other evidence has emerged that anesthetics can also induce neuroinflammation, impair hippocampal synaptic plasticity, and cause neurodegenerative changes, indicating the possible detrimental effects of anesthetics on both the young developing and the elderly aging brain (Wan et al., 2021; Yang et al., 2021; Rump and Adamzik, 2022). Still, some experimental evidence of potential neuroprotective effects has been reported, making it somewhat equivocal. Therefore, further preclinical research is needed to enrich our understanding of anesthetics. In addition, it usually combines several anesthetics rather than a single anesthetic in perioperative anesthesia management, especially major surgeries and time-consuming surgeries. For instance, dexmedetomidine combined with propofol may decrease the demand doses and side effects of sedation (Elbakry and Ibrahim, 2017; Mason et al., 2021). We suggest that researchers pay attention to the adverse risks of anesthesia regimens when exploring the potential beneficial effects of synergistic use of anesthetics on intestinal IRI treatment. Developing appropriate anesthesia plans based on the patient’s condition is essential for achieving safe medication.

**FIGURE 1:**
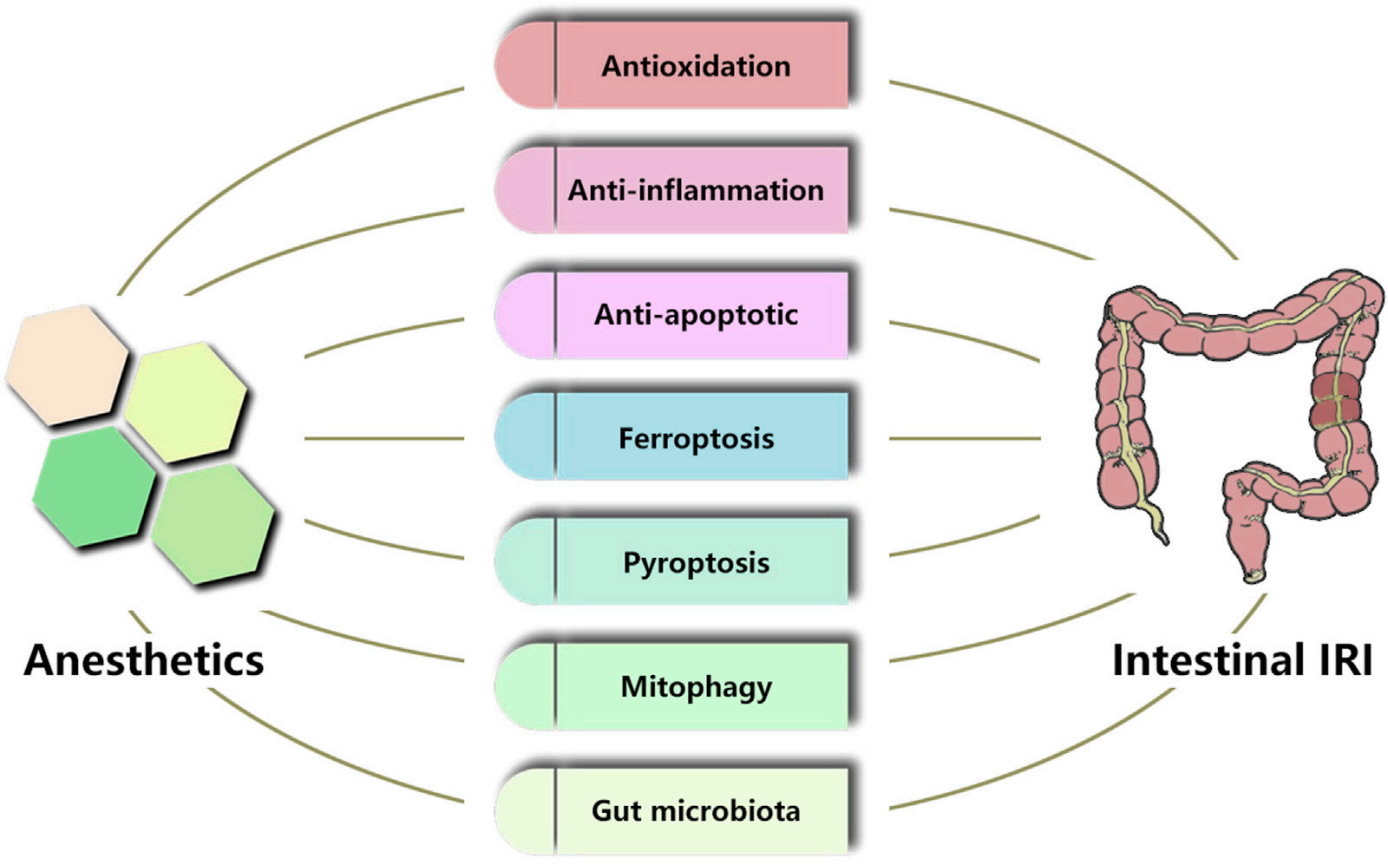
The possible mechanisms of protection effects of anesthetics in intestinal IRI.

Third, we have learned that pre-treatment of specific anesthetics may provide outstanding protection effects for intestinal IRI and ALI from the introduced research in this review. Most studies confirmed that antioxidation, anti-inflammation, and anti-apoptotic are possible mechanisms of these protection effects. In recent years, scholars have also found that anesthetics regulate ferroptosis, pyroptosis, mitophagy, and gut microbiota to protect the intestinal mucosa (Figure 1). However, the exact mechanism of intestinal IRI is still unclear (Zhang et al., 2023a). Intestinal IRI causes injury to multiple extraintestinal organs and is a multi-step process involving intestinal flora disturbance, bacterial translocation, endotoxin release, and multiple signaling pathways are involved (Chen et al., 2021a; Li et al., 2021b; Deng et al., 2023). Some scholars are also committed to revealing the pathological mechanisms of intestinal IRI from new perspectives. Non-coding RNAs, extracellular vesicles (EVs), intestinal microbiota derivatives, complement activation, and neutrophil extracellular traps play essential roles in the pathogenesis of intestinal IRI (Chen et al., 2020b; Chen et al., 2021b; Wu et al., 2021; Zhang et al., 2023a; Zhang et al., 2023b). These findings will also further drive scholars to explore the mechanism of anesthetics to protect against intestinal IRI and help refine our knowledge in this aspect.

In a word, our review shows the beneficial effects of anesthetics on intestinal IRI, indicating that reasonable anesthetic management in the perioperative period may be an important strategy for avoiding intestinal IRI in major surgical procedures. Although current related research is still focused on preclinical studies, we should encourage further research efforts in this direction.
